# Supplementary material for: Cross-modal fusion of brain imaging and clinical data for Parkinson’s disease progression prediction
Source: PLoS One. 2025 Nov 20;20(11):e0333822. doi: 10.1371/journal.pone.0333822 (PMC12633903; doi:10.1371/journal.pone.0333822)
Supplement: Attachment — Submitted filename: Response to Reviewers.docx [file pone.0333822.s001.docx]

Cross-modal fusion of brain imaging and clinical data for Parkinson's disease progression prediction
Response to the review comments

Dear Editors of PLOS ONE,

We have revised the paper “Cross-modal fusion of brain imaging and clinical data for Parkinson's disease progression prediction” (Previous I.D.: PONE-D-25-29378) according to the reviewer’s comments and suggestions, further enhanced the text, smoothed the presentation, and proofread the revised paper to ensure that there are no typos. In what follows, we present our replies to the reviewer’ comments/suggestions one by one (our replies are colored in blue). We thank all the editors and reviewers, and their valuable detailed comments. They are very helpful to us!

Thank you for the time and efforts! Please let us know if any further revision is needed.

Sincerely,

Jinyu Wen, Amei Chen, Jingxin Liu, Hua Xiong, Meie Fang, Xinhua Wei

-------------------------------------------------------------------------------------------------------

When submitting your revision, we need you to address these additional requirements.
1. Please ensure that your manuscript meets PLOS ONE's style requirements, including those for file naming. The PLOS ONE style templates can be found at https://journals.plos.org/plosone/s/file?id=wjVg/PLOSOne_formatting_sample_main_body.pdf and
<https://journals.plos.org/plosone/s/file?id=ba62/PLOSOne_formatting_sample_title_authors_affiliations.pdf>

2. Please note that PLOS One has specific guidelines on code sharing for submissions in which author-generated code underpins the findings in the manuscript. In these cases, we expect all author-generated code to be made available without restrictions upon publication of the work. Please review our guidelines at https://journals.plos.org/plosone/s/materials-and-software-sharing#loc-sharing-code and ensure that your code is shared in a way that follows best practice and facilitates reproducibility and reuse.

Our response 1-2:

Thanks for the reviewer’s encouraging remarks and valuable comments. The format has been improved according to the requirements of the journal and the code has also been uploaded to [Dryad Digital Repository](https://datadryad.org/) (DOI: [10.5061/dryad.63xsj3vdv)](https://doi.org/10.5061/dryad.63xsj3vdv).), which can be found at
http://datadryad.org/share/zvyCwl-SD-ApTC7IwAXSLJJxA1W53rjXR_c1xPykaxM.

3. We note that the grant information you provided in the ‘Funding Information’ and ‘Financial Disclosure’ sections do not match.

When you resubmit, please ensure that you provide the correct grant numbers for the awards you received for your study in the ‘Funding Information’ section.

4. Thank you for stating the following financial disclosure:
“This work was supported in part by the National Natural Science Foundation of China (No. 62072126), in part by the Fundamental Research Projects Jointly Funded by Guangzhou Council and Municipal Universities No. SL2023A03J00639, in part by the Key Laboratory of Philosophy and Social Sciences in Guangdong Province of Maritime Silk Road of Guangzhou University (GD22TWCXGC15), in part by the Natural Science Foundation of Chongqing (No. CSTB2024NSCQ-MSX1087), and the Guangxi Science and Technology Program (No.AD23023001).”
Please state what role the funders took in the study. If the funders had no role, please state: "The funders had no role in study design, data collection and analysis, decision to publish, or preparation of the manuscript."

If this statement is not correct you must amend it as needed.

Please include this amended Role of Funder statement in your cover letter; we will change the online submission form on your behalf.

5. Thank you for stating in your Funding Statement:

“This work was supported in part by the National Natural Science Foundation of China (No. 62072126), in part by the Fundamental Research Projects Jointly Funded by Guangzhou Council and Municipal Universities No. SL2023A03J00639, in part by the Key Laboratory of Philosophy and Social Sciences in Guangdong Province of Maritime Silk Road of Guangzhou University (GD22TWCXGC15), in part by the Natural Science Foundation of Chongqing (No. CSTB2024NSCQ-MSX1087), and the Guangxi Science and Technology Program (No.AD23023001).”
Please provide an amended statement that declares *all* the funding or sources of support (whether external or internal to your organization) received during this study, as detailed online in our guide for authors at http://journals.plos.org/plosone/s/submit-now.  Please also include the statement “There was no additional external funding received for this study.” in your updated Funding Statement.
Please include your amended Funding Statement within your cover letter. We will change the online submission form on your behalf.

6. Thank you for stating the following in the Acknowledgments Section of your manuscript:
   “This work was supported in part by the National Natural Science Foundation of China (No. 62072126), in part by the Fundamental Research Projects Jointly Funded by Guangzhou Council and Municipal Universities No. SL2023A03J00639, in part by the Key Laboratory of Philosophy and Social Sciences in Guangdong Province of Maritime Silk Road of Guangzhou University (GD22TWCXGC15), in part by the Natural Science Foundation of Chongqing (No. CSTB2024NSCQ-MSX1087), and the Guangxi Science and Technology Program (No.AD23023001).”

We note that you have provided funding information that is not currently declared in your Funding Statement. However, funding information should not appear in the Acknowledgments section or other areas of your manuscript. We will only publish funding information present in the Funding Statement section of the online submission form.

Please remove any funding-related text from the manuscript and let us know how you would like to update your Funding Statement. Currently, your Funding Statement reads as follows:

“This work was supported in part by the National Natural Science Foundation of China (No. 62072126), in part by the Fundamental Research Projects Jointly Funded by Guangzhou Council and Municipal Universities No. SL2023A03J00639, in part by the Key Laboratory of Philosophy and Social Sciences in Guangdong Province of Maritime Silk Road of Guangzhou University (GD22TWCXGC15), in part by the Natural Science Foundation of Chongqing (No. CSTB2024NSCQ-MSX1087), and the Guangxi Science and Technology Program (No.AD23023001).”

Please include your amended statements within your cover letter; we will change the online submission form on your behalf.

Our response 3-6:

Thank you very much for your very kind suggestions. During the process of submitting research materials through the system, we provided the correct funding numbers and revised the funding statement accordingly.

7. Please include a separate caption for each figure in your manuscript.

Our response 7:

Thanks for your detailed suggestions. I have added individual captions for each figure.

**Reviewer #1:**

1.Clinical Information of Study Subjects

Please provide a demographic table summarizing the clinical characteristics of the Parkinson’s disease patients included in this study. In particular, information for both the HYS deterioration group and the non-deterioration group at baseline and at the 5-year follow-up is necessary to allow for a clearer understanding of cohort composition and comparability.

Our response:

Thanks very much for your valuable comments! I have already uploaded a demographic table (demographic-clinical_characteristics_HYS.xlsx) into [Dryad Digital Repository](https://datadryad.org/) (http://datadryad.org/share/zvyCwl-SD-ApTC7IwAXSLJJxA1W

53rjXR_c1xPykaxM). This table includes several key clinical characteristics that have been screened from the Parkinson's disease patients included in this study. It also includes comparative data on disease progression (such as HYS scores) at baseline and during 5-year follow-up.

2. Clinical Significance of HYS Changes at Different Stages

This study categorizes patients based on the presence or absence of Hoehn and Yahr Scale (HYS) progression. However, the clinical significance of changes in HYS scores depends heavily on the specific stages involved. For example, a change from stage 1 to 2 may simply reflect a transition within the early, so-called “honeymoon” phase, while a shift from stage 2 to 3 typically indicates entry into the more progressive stage of the disease. I recommend addressing this issue in the discussion or including it as part of the limitations of the study.

Our response:

Thanks very much for your valuable comments! The clinical importance of changes in the HYS exhibits significant stage-dependent characteristics. We fully agree that there is a clear distinction between the progression from Stage 1 to Stage 2 (which typically reflects the worsening of unilateral symptoms or the emergence of mild symptoms on the contralateral side) and the progression from HYS Stage 2 to Stage 3 (which marks the onset of impaired postural reflexes and balance disorders, serving as a critical turning point indicating the disease's advancement to a more severe stage). We have carefully considered this suggestion and taken the following measures in the revised manuscript.

A dedicated paragraph has been added to the discussion section: It explicitly acknowledges that the clinical significance of HYS changes is highly dependent on the specific stage at which they occur. Particular emphasis is placed on the unique importance of the transition from HYS Stage 2 to Stage 3, describing it as a crucial clinical milestone in disease progression that typically heralds greater disability, an increased risk of falls, and changes in treatment response. In contrast, it is clarified that while changes from Stage 1 to Stage 2 of HYS indeed indicate symptom progression, their impact on functional status and prognosis is generally less profound than the transition from Stage 2 to Stage 3, and often occurs during the relatively early "honeymoon period."

We discuss how these stage-dependent differences may influence the interpretation of the findings of our study. For example, if the study cohort includes a larger proportion of patients experiencing early-stage (stages 1-2) changes, the "clinical weight" of their progression may be lower than that of a cohort with more stages 2-3 transitions. We will carefully elaborate on the potential impact of this factor on the generalizability and effect size of our study findings.

We have also supplemented a limitation: "Although HYS changes vary in clinical significance across stages, this study did not distinguish the exact stage of progression."

3. Limitations of HYS as an Assessment Scale

The HYS is a relatively coarse measure of disease severity. In clinical practice, many PD patients are classified as stage II, but the degree of motor symptom severity within this group can vary widely. Therefore, many previous studies use MDS-UPDRS Part III scores as a more granular and sensitive indicator of motor function. Please consider discussing this limitation of the HYS in light of the metrics used in prior literature, particularly as it relates to your classifier’s outcome and feature selection.

Our response:
Thank you very much for your very kind suggestions. This points out the inherent limitations of the HYS scale in terms of resolution and sensitivity, particularly highlighting the high heterogeneity within the HYS stage II patient group. In response to this suggestion, we have made the following modifications in the revised manuscript to address it adequately:

Added a new paragraph in the Discussion section: We explicitly acknowledge the roughness of the HYS scale, especially its inability to capture subtle changes in motor function at Stage II. In contrast, we elaborate on the advantages of the MDS-UPDRS Part III: As a continuous variable scale, it can provide more sensitive and objective monitoring of disease progression by quantifying specific motor signs (such as tremor, rigidity, and bradykinesia)[36].

We explain the rationale for selecting the HYS scale in this study. Given that the research objective focuses on transitions in disease stages (for example, the critical turning point from Stage II to Stage III), and the HYS scale, as the gold standard for clinical staging, exhibits a stronger correlation with long-term prognosis while facilitating standardized data collection in multicenter studies [37].

Added new statements in the Limitations section: "Although HYS changes vary in clinical significance across stages, this study did not distinguish the exact stage of progression. Although the selection of the HYS scale as a marker of disease progression in this study offers the advantage of universal applicability in clinical staging, its insufficient resolution may weaken the analysis of the correlation with continuous changes in motor function. Future studies need to combine fine - grained scales such as the MDS - UPDRS to validate the generalization ability of this classifier."

References used in the response to the revised manuscript:

[36] Skorvanek M, Martinez‐Martin P, Kovacs N, et al. Differences in MDS‐UPDRS scores based on Hoehn and Yahr stage and disease duration[J]. Movement disorders clinical practice, 2017, 4(4): 536-544.

[37] Ygland Rödström E, Puschmann A. Clinical classification systems and long-term outcome in mid-and late-stage Parkinson’s disease[J]. npj Parkinson's Disease, 2021, 7(1): 66.

4. Comparison with Previous Studies

While this study proposes a novel machine learning classifier to predict PD progression, there is a large body of existing literature with similar goals. However, the current manuscript lacks a comparative analysis of the proposed model’s performance relative to those of prior studies. I strongly recommend including a discussion of how your model’s AUC and other evaluation metrics compare to previously published classifiers, to contextualize the novelty and value of your findings.

Our response:

Thanks very much for your valuable comments! To fully substantiate the effectiveness of the proposed method, we have supplemented a table in the results section (as shown in Table 5 of the revised manuscript). This table mainly incorporates several new works on PD progression prediction for comparative analysis. The methods in the table primarily focus on research on the combined application of multimodal data, covering several types: the combination of clinical and genetic data, the combination of clinical and imaging data, the combination of clinical, genetic and imaging data, and the combination of clinical, biomarker, and imaging data. Most existing studies employ traditional machine learning methods, while we have put forward a new method, which reflects the innovative nature of the method presented in this paper. From a comprehensive analysis of the evaluation metric values, overall, our method demonstrates relatively significant effectiveness in predicting the 5-year progression of PD.

5. Rationale for Feature Selection

Although clinical features were selected using the Lasso method, the imaging modalities (DTI and DAT SPECT) were chosen a priori. The rationale for restricting imaging feature selection to DTI and DAT SPECT is not clearly explained. Given that many previous studies have employed structural features extracted from 3D T1-weighted images—such as brain volume and cortical thickness—it would be worth discussing whether incorporating such features could potentially improve classifier performance.

Our response:

Thanks for your detailed suggestions. Regarding the selection of imaging modalities, we have supplemented our discussion section with additional clarifications. Our decision to focus on DTI and DAT as primary neuroimaging assessment tools to predict progression of PD was based on multiple scientific considerations. From a pathophysiological perspective, DTI offers unique insights into white matter microstructural integrity. By measuring the diffusion anisotropy of water, DTI precisely characterizes the orientation and structural integrity of the nerve fiber bundles. This technical advantage is particularly crucial for understanding PD, a quintessential neurodegenerative disorder, as the accumulation of evidence shows that microstructural damage to white matter plays a pivotal role in the development of motor and non-motor symptoms.

Furthermore, DAT imaging provides clinically irreplaceable value. This technique enables visualization of the spatial distribution and functional status of the dopamine transporter, directly evaluating the integrity of the nigrostriatal dopaminergic system. Given that dopaminergic dysfunction constitutes the main pathological hallmark of PD, quantitative indices derived from DAT imaging show robust correlations with disease severity and progression stages.

Although structural features extracted from 3D T1-weighted images (e.g., brain volume and cortical thickness) undoubtedly hold significant value in PD research by reflecting morphological brain changes and contributing to diagnostic assessment, these structural alterations typically manifest later in disease progression compared to the white matter microstructural changes detected by DTI and the functional abnormalities identified through DAT imaging. This temporal consideration led to our decision to exclude 3D T1-weighted imaging from the analytical framework of the current study.

**Reviewer #2:**

Reviewer 2: The authors aimed to develop a model based on MRI/DTI scans to provide a reliable biomarker useful for prediction of PD at its early stage. With the PPMI database of multi-center PD patients, the proposed cross-modality fusion prediction method (CMFP) appears superior in PD-progression prediction performance compared to single-modality approach. Machine learning is an increasing promising tool for clinic diagnosis; however, the comparative predictive values of its use combined with MRI/DTI remain unclear. In this sense, the manuscript provided a badly needed model to this end. I’ve several major and minor concerns regarding this manuscript (see below).

Major concerns:

1. The authors reported their results in a very casual way. They basically skipped the reporting of the Figure 4 and the Figure 5 in the Results (lines 260), which was then brought into a detailed description in the Discussion. As a result, the Discussion session was mixed with results and discussion;

Our response:

Thanks very much for your valuable comments! The original intention of Figure 4 is to discuss the predictive effects of single-modality and multimodality combinations using ROC curves, thus demonstrating the necessity of the strategy proposed in this paper. Figure 5, on the other hand, performs a comparative analysis of AUC and ROC by replacing the decision fusion strategy with different traditional machine learning algorithms, in order to prove the effectiveness of the method proposed in this paper and further confirm that the combination of clinical data and DTI yields better results in predicting the progression of PD. To enhance the readability of the article, we have provided a more detailed analysis and explanation of Figure 4 and Figure 5, presenting them as a separate paragraph.

2. It’s very confusing that it appears the CMFP is the prediction model the authors were trying to sell, yet CMFP was rarely seen throughout the manuscript (not once in the Discussion). In contrast, the authors focused on the Decision Fusion Predictive Model (line 96);

Our response:

Thank you very much for your very kind suggestions. In this work, we have proposed a PD progression prediction model, named CMFP, which is essentially a "Decision Fusion Predictive Model". The general framework of this model consists of four parts: data pre-processing, feature selection, classifier modeling, and prediction. Among the prediction methods, the decision-making strategy CMF is a crucial component. In Section 3.1, through comparative experimental results, we have analyzed the role played by CMF and demonstrated the effectiveness of the proposed prediction model. The "Ours" method mentioned in the discussion section refers to CMFP. To make the methods in the paper more comprehensible, we have made some modifications. For example, we added the abbreviation of the method in the title of Section 2.2 and described the CMF process in the last paragraph of Chapter 2. Additionally, we have replaced 'ours' in the figure with the abbreviation of CMFP.

3. Many multi-modal prediction models exist as the author mentioned (refs 25-29). The rationale and mechanisms the CMFP were superior to other models is not clear in this manuscript. Have the authors applied these models on the PPMI dataset used in this study? Have they authors applied their CMFP model to other PD dataset(s)? The comparison was missing in the Results. But at least these should be mentioned in the Discussion.

Our response:

Thanks for your detailed suggestions. This work has only been validated on publicly available datasets(PPMI) and lacks validation with external PD data. This represents a limitation of our study, which we have supplemented and explained in the conclusion section of the revised manuscript. To fully substantiate the effectiveness of the proposed method, we have supplemented a table in the results section (as shown in Table 5 of the revised manuscript). This table mainly incorporates several new works on PD progression prediction for comparative analysis. The methods in the table primarily focus on research on the combined application of multimodal data, covering several types: the combination of clinical and genetic data, the combination of clinical and imaging data, the combination of clinical, genetic and imaging data, and the combination of clinical, biomarker, and imaging data. Most existing studies employ traditional machine learning methods, while we have put forward a new method, which reflects the innovative nature of the method presented in this paper. From a comprehensive analysis of the evaluation metric values, overall, our method demonstrates relatively significant effectiveness in predicting the 5-year progression of PD.

Minor concerns:

1. The writing and organization of the manuscript is chaotic, specifically: 1) the long passage (lines 16-51) in the Introduction session is better to split into two parts, 2) using the bullet points in the Introduction is discouraged, 3) Python and Pytorch should be in the Methods session (lines 199-200);

2. The English/grammar need to be improved/corrected significantly throughout the manuscript. It’s difficult to read and follow. For example, the result summary titles (the lines 206, 248) were illy composed. In the line 359, “AUC used to measure the model’s classification ability.” And many more throughout the text and legends;

3. The statistically significance level was not specified. What main statistic method was used for group comparison (other than Mann-Whitney U test)? How was the statistics performed? How were the age and sex controlled?

4. In the Table 5, full names of SEN and SPE should be used. No need to abbreviate;

5. Remove the numbering in the Methods and Results sessions;

6. Remove the redundant abbreviation of PD in the line 274;

7. The manuscript images seem fuzzy and hard to read. Were the authors using appropriate dpi?

Our response 1-7:

Thank you very much for your very kind suggestions. To enhance the readability of the paper, we have made corresponding adjustments in the revised manuscript:

(1)The content from lines 16 to 51 in the introduction section has been split into two paragraphs; the bullet points previously used have been replaced with numerical numbering; and the descriptions regarding Python and Pytorch, etc., have been moved to the methods section.
(2)The two headings in the results section have been adjusted and sentences such as "AUC used to measure the model's classification ability" have been revised to "The AUC metric was employed to quantitatively assess the model's discriminative performance".
(3) In the statistics and discussion sections, we use the Mann-Whitney U test to compare the performance between clinical data prediction alone and clinical DTI integrated prediction, with a significance level set at α=0.05. The results demonstrated that the fusion model significantly outperformed the clinical-only model (p=9.183×10^-4). All statistical analyzes were implemented using Python programming. It should be noted that while this study has certain limitations in controlling for age and gender factors, the significance comparison of prediction outcomes still validated the model's effectiveness.
(4)The abbreviations "SEN" and "SPE" in the table have been replaced with their full names.
(5)The numbering in the "Methods" and "Results" sections has been removed.
(6)The full form of "PD" in line 274 of the original manuscript has been removed, with only the abbreviation retained.
(7)The resolution of the images has been improved for easier reading and comprehension.

**Reviewer #3:**

Reviewer 3: Researchers attempted to predict Parkinson's disease progression using three data modalities (clinical, DTI imaging, DaTscan) combined through cross-modal fusion to improve upon single-modality predictions. Although the methodology appears rigorous, fundamental questions remain unanswered.

1. Serious methodological problems:

i. Training for 120 epochs with only 123 total patients (even fewer after data splitting) will inevitably cause the model to memorize training examples rather than learn generalizable patterns. Why was such high epoch used?

ii. How is AdaBoost integrated with Adam optimizer? AdaBoost operates through weighted voting without gradient descent, while Adam is specifically designed for gradient-based neural network optimization - these approaches are fundamentally incompatible.

iii. How is a classification model using mean squared error loss? Classification problems require cross-entropy loss, not regression losses like MSE that are designed for continuous value prediction.

iv. Feature selection performed before data splitting is fundamentally wrong and promotes data leakage, where the selection process "sees" test data and artificially inflates performance estimates.

v. There is a sample bias between positive and the negative class. Was the split stratified?

Our response 1:

Thanks very much for your valuable comments!

i. We recognize that training for an excessive number of epochs may increase the risk of overfitting, especially given the limited sample size. Therefore, we have mitigated this issue through the following strategies: For instance, we employed a learning rate scheduler, reducing the learning rate to 0.1 times its previous value every 30 epochs to prevent drastic parameter oscillations in the later stages of training. We also applied L2 regularization to indirectly suppress overfitting. Additionally, we adopted 8-fold cross-validation to make full use of the limited sample data. These strategies help ensure that the model learns the true underlying data patterns rather than merely memorizing the training samples.

ii. The compatibility issue raised by the reviewers is indeed crucial. However, in our current model implementation, AdaBoost serves solely as a base classifier in the feature extraction stage, where its role is to generate individual prediction probabilities for each modality (e.g., clinical and DTI modalities). The proposed cross-modal fusion model, which is trained independently using the Adam optimizer, merely takes AdaBoost's outputs as input features. The entire optimization process of the fusion network relies exclusively on the Adam optimizer, ensuring no direct dependency on AdaBoost's internal optimization mechanism.

iii. We fully agree that the cross-entropy loss function is typically the standard choice for classification tasks. In this study, however, the prediction target is the PD progression score (e.g., 1, 0), which represents an ordered discrete variable. Our decision to use MSE as the loss function was primarily based on the following two considerations: First, MSE imposes a smoother penalty on deviations in continuous probability, a characteristic that may help mitigate class imbalance issues to some extent. Second, in the fusion prediction of the two modalities, the labels incorporate the predicted probabilities from each modality. Compared to other loss functions, MSE may be more effective in optimizing the fusion weights.

iv. We fully concur with the reviewer's critique that performing feature selection prior to data partitioning would lead to data leakage, where the selection process could inadvertently "peek" at test set information and consequently bias performance evaluation. Our study rigorously adheres to machine learning best practices: All feature selection procedures were performed exclusively within the training set, while the test set solely for final validation purposes. We have accordingly revised the CMFP workflow in the Methods section (Step 2) to explicitly delineate the boundary between feature selection and model training, thereby ensuring our experimental design is free from any data leakage risks.

v. We acknowledge that class imbalance may affect model performance. In this study, the dataset was partitioned by randomly shuffling indices without employing stratified sampling. While this approach could potentially lead to model bias toward the majority class and reduce minority class recognition, we implemented specific countermeasures in the evaluation design: First, we adopted AUC as the primary metric given its relative insensitivity to class distribution skew, which helps mitigate evaluation bias from imbalance. Second, we conducted eight independent data partitioning experiments to verify model stability. Although explicit stratified sampling was not used, the experimental results (as shown in revised Table 6 and Figure 6) demonstrate that the model maintains reasonable performance in both sensitivity and specificity metrics, indicating the current approach partially alleviates the negative impact of class imbalance. In future work, we will further optimize the data partitioning strategy by incorporating stratified sampling to more comprehensively improve minority class recognition capability.

2. Other problems:

i. Variables in equations 1-6 (ρ, ψ, τ, μ, ϕ, λ, ι, α, υ) lack proper definition - what do these variables represent and how do they relate to the fusion process?

ii. No nested cross-validation, no external validation, confidence intervals missing.

iii. Authors show only one p-value which signifies one statistical test but the authors comment about multiple tests being performed. Where is the multiple comparison test?

iv. Claims such as novel CMF without proper citations. There are multiple claims without citations.

Our response 2:

Thanks for your detailed suggestions.

i. The variables in the formula have been supplemented with explanations.

ii. This work used 8-fold cross-validation without external validation, which is indeed a limitation that has now been explicitly acknowledged in the conclusion section. We will pay closer attention to this aspect in future work and strive to collect additional datasets for external validation purposes.

iii. As this study primarily proposes a novel network model, the focus is predominantly on evaluating the performance of the model. In terms of statistical analysis, we calculated p-values and examined variances across evaluation metrics. Specifically, in the statistical analysis and discussion sections, we employed the Mann-Whitney U test to compare the performance between the prediction of clinical data only and the prediction of clinical-DTI fusion. The results show that the fusion model significantly outperforms the clinical only model (p = 9.183×10^-4), indicating that the proposed network model achieves statistically significant improvements.

iv. The CMF algorithm has been added to the methodology section, and citations have been supplemented in multiple places.

3. Grammar:

i. Poor grammar encountered numerous times in the paper.

ii. They write "using imaging-omics, particularly clinical data" which is contradictory since clinical data typically isn't considered part of imaging-omics.

iii. The authors inconsistently refer to their method as "CMFP," "CMF," and "cross-modal fusion" without clearly establishing these as equivalent terms.

iv. Claims like "DTI metric along the perivascular space has relatively more advantages" is vague without specifying compared to what.

Our response 3:

Thank you very much for your very kind suggestions.

i. General questions have been addressed in the revised manuscript.

ii. The original phrasing "using imaging-omics, particularly clinical data" was indeed inappropriate and has been modified to "using imaging and clinical data".

iii. CMF (cross-modal fusion) refers specifically to our strategy to merge heterogeneous modalities. CMFP (cross-modal fusion prediction) represents our complete predictive model. Note that CMF constitutes a core component of CMFP. We have added explanatory details in the Methods section to improve readability.

iv. The original description was indeed ambiguous. We have clarified the comparative advantage by modifying the sentence to: "Using the DTI metric along the perivascular space (DTI-ALPS) to predict and evaluate the progression of PD has relatively more advantages compared to DTI-clinical fusion prediction. Among them, the ACC can increase by 3.85%."

The reviewer’s comments and suggestions have been carefully addressed in the revised version of the paper. Changes in the revised version have been colored red. We are grateful once again to the associate editor and reviewers for their time and helpful comments, which have played a vital role in improving the quality and presentation of the original manuscript.
