# Supplementary material for: Cross-modal fusion of brain imaging and clinical data for Parkinson’s disease progression prediction
Source: PLoS One. 2025 Nov 20;20(11):e0333822. doi: 10.1371/journal.pone.0333822 (PMC12633903; doi:10.1371/journal.pone.0333822)
Supplement: Attachment — Submitted filename: Response_to_Reviewers_auresp_2.docx [file pone.0333822.s002.docx]

Cross-modal fusion of brain imaging and clinical data for Parkinson's disease progression prediction
Response to the review comments

Dear Editors of PLOS ONE,

We have revised the paper “Cross-modal fusion of brain imaging and clinical data for Parkinson's disease progression prediction” (Previous I.D.: PONE-D-25-29378) according to the reviewer’s comments and suggestions, further enhanced the text, smoothed the presentation, and proofread the revised paper to ensure that there are no typos. In what follows, we present our replies to the reviewer’ comments/suggestions one by one (our replies are colored in blue). We thank all the editors and reviewers, and their valuable detailed comments. They are very helpful to us!

Thank you for the time and efforts! Please let us know if any further revision is needed.

Sincerely,

Jinyu Wen, Amei Chen, Jingxin Liu, Hua Xiong, Meie Fang, Xinhua Wei

-------------------------------------------------------------------------------------------------------

Reviewer #3: Thanks to the authors for the changes. Some of the problems were adequately answered but the paper remains confusing.

**Major:**

1. Single-modality AUC values (0.40-0.50) are worse than random chance, indicating a methodological issue.

Our response:

Thanks very much for your valuable comments! The issue you pointed out regarding the low single-modality AUC values (ranging from 0.40 to 0.50) is indeed crucial, and we fully agree that it warrants in-depth discussion. The fact that the AUC values for single-modality prediction results are below random levels does not stem from methodological errors or flaws in code implementation. Instead, it profoundly reveals the inherent limitations of single-modality data in addressing such complex biomedical problems. Certain single data perspectives (such as individual clinical scales or single imaging features) may inherently lack sufficient information to distinguish disease states and could even contain substantial noise or confounding factors unrelated to the true biological signals. When the model learns based on such information, its weak and unstable predictive signals can cause performance to fluctuate around the level of random guessing. The core of this study is precisely aimed at addressing this dilemma. The experimental results (as shown in Table 4 and Figure 4) demonstrate that the performance of single-modality models is significantly and consistently inferior to that of multimodal fusion models, highlighting the necessity of fusing multi-source information. The single-modality baselines, serving as negative controls, effectively demonstrate the advantages of our proposed method. In accordance with your suggestion, we have added further explanation of this phenomenon in the Results section of the paper.

2. Patient number mismatch: "…it was found that 74 patients had scores higher than baseline…" vs "…progression group (n = 72)…".

Response:

Thank you very much for your thorough review of our paper and the valuable feedback you provided. Upon verification, there was indeed an error in the reported number of patients. The progression group should include 74 cases, and this has been corrected in the manuscript to ensure data accuracy.

3. Missing confidence intervals for AUC values in Tables 2, 4, and 6. With reported variance of 0.1085, and without confidence intervals the reliability of the 0.7791 AUC cannot be assessed.

Response:

Thanks very much for your valuable comments! The results presented in Tables 2, 4, and 6 consistently demonstrate that the performance of the multimodal fusion prediction model significantly outperforms that of single-modality prediction models, effectively enhancing predictive accuracy. Among the multimodal fusion strategies, the combination of clinical data and DTI imaging exhibited particularly outstanding performance, achieving the best predictive outcomes. As shown in Figure 4, we systematically compared the discriminative abilities of various models using ROC curves and AUC confidence intervals. Figure 4(a) displays the ROC curves of different models, where it can be visually observed that the curves of multimodal fusion models (especially the clinical+DTI combination) are closer to the top-left corner, indicating superior classification performance. Figure 4(b) further presents the confidence intervals of the AUC values for each model, providing statistical evidence for the stability of model performance. Specifically, the fusion of clinical and DTI data achieved an AUC of 0.7719, with a 95% confidence interval of (0.67-0.86), demonstrating both high discriminative power and robust results. In contrast, the AUC confidence intervals for single-modality predictions were lower, generally approaching or below random chance levels, with wider fluctuations indicating unstable performance. Additionally, the absence of overlapping confidence intervals between single-modality and multimodal prediction models further statistically validates the significant superiority of the multimodal fusion approach. These results collectively indicate that integrating clinical and DTI information significantly enhances predictive performance, with the advantage being statistically significant. The limited information available to single-modality models leads to poorer performance and higher uncertainty, further underscoring the necessity and effectiveness of multimodal fusion strategies.

4. High performance variance (0.1085) indicates unstable results but no discussion of reproducibility implications.

Response:

Thanks very much for your valuable comments! We fully agree that the high variance in performance metrics (0.1085) is indeed a crucial consideration for evaluating model robustness and reproducibility. After thorough analysis, we believe this variance primarily stems from the following two reasons: On one hand, the dataset in this study has a limited sample size (n=123) with high clinical heterogeneity among patients. In such small-sample, high-heterogeneity data, different training-test splits can reasonably lead to some degree of performance fluctuation. On the other hand, our evaluation using 8-fold cross-validation inherently reflects the model's sensitivity to different data subsets; this variance does not indicate a model flaw but rather provides an objective measure of uncertainty regarding its generalization capability. Despite the observed variance, the main conclusions of this study remain robust. As shown in Figure 4, the performance of our proposed multimodal fusion approach significantly outperforms all single-modality baselines. Particularly compared to using clinical data alone, the fusion of clinical and DTI data demonstrates substantial improvement (p = 9.183e-4), indicating clear statistical significance and reliable conclusions. We sincerely appreciate the reviewer's valuable comment and have accordingly supplemented the discussion section of the paper with an analysis regarding the implications of performance variance.

**Minor:**

1. CMFP/CFMP, CMF/CFM used interchangeably.

Our response:
Thank you very much for your kind suggestion. The abbreviations CMFP and CMF are defined based on the initial capitalization sequence of the method names. For instance, CMFP is derived from "Cross-Modal Fusion Prediction Model," while CMF comes from "Cross-Modal Fusion." Therefore, replacing CMFP with CFMP and CMF with CFM would not be appropriate.

2. "Multivariate regression analysis further confirmed a negative…" missing a citation.

Response:

Thank you for your meticulous suggestion. The statement "multivariate regression analysis further confirmed the negative..." in the paper has now been supplemented with the corresponding citation.

3. ACC, AUC, ESS, RBDSQ, UPSIT, etc undefined in introduction. Please provide a table for all the abbreviations.

Response:

Thank you very much for your kind suggestion. The full forms of abbreviations such as ACC, AUC, ESS, RBDSQ, and UPSIT are all provided at the end of the paper, as shown in Table 7.

4. In table 2 ' tau i' should explicitly mention that 'i' is just the corresponding 'i' as given in table 3. Otherwise it looks confusing. Or present table 3 before 2.

Response:

Thank you for your meticulous suggestion. To provide a more intuitive and understandable presentation, Table 3 has been placed before Table 2.

5. "The clinical significance of HYS…" the first three paragraphs of discussion, present claims without citations.

Response:

Thanks very much for your valuable comments! The clinical significance of HYS has been supplemented with corresponding citations in the first paragraph of the Discussion section.

6. "four clinical data" in methods should be "four clinical variables" and other grammatical errors throughout.

Response:

Thank you very much for your kind suggestion. Grammatical errors such as "four clinical data" and "Several researches have" have been corrected as shown in the annotated manuscript.

7. "Larger sample size for improved prediction" in existing methods comparison. Larger sample size reduces variance but doesn't guarantee better performance - Liu et al. (citation 25) achieved higher AUC/ACC scores with only 33 PD patients.

Response:

Thanks very much for your valuable comments! The statement "Larger sample size for improved prediction" was indeed inappropriately phrased. While a large sample size may enhance predictive performance in the methodology of this study, this may not necessarily hold true for other approaches. Accordingly, I have revised it to: "which was supported by a large sample size to enhance statistical power and generalizability. It should be noted, however, that sample size alone does not determine predictive accuracy, as demonstrated by Liu et al."

The reviewer’s comments and suggestions have been carefully addressed in the revised version of the paper. Changes in the revised version have been colored red. We are grateful once again to the associate editor and reviewers for their time and helpful comments, which have played a vital role in improving the quality and presentation of the original manuscript.
